# Alleged Approach-Avoidance Conflict for Food Stimuli in Binge Eating Disorder

**DOI:** 10.1371/journal.pone.0152271

**Published:** 2016-04-05

**Authors:** Elisabeth J. Leehr, Kathrin Schag, Amelie Brinkmann, Ann-Christine Ehlis, Andreas J. Fallgatter, Stephan Zipfel, Katrin E. Giel, Thomas Dresler

**Affiliations:** 1 Department of Psychosomatic Medicine and Psychotherapy, University Hospital Tuebingen, Internal Medicine VI, Tuebingen, Germany; 2 Department of Psychiatry and Psychotherapy, University Hospital Tuebingen, Tuebingen, Germany; 3 LEAD Graduate School, University of Tuebingen, Tuebingen, Germany; 4 Department of Medicine and Psychosomatics, Centre for Psychosocial Medicine, University Hospital Heidelberg, Heidelberg, Germany; Tsinghua University, CHINA

## Abstract

**Objective:**

Food stimuli are omnipresent and naturally primary reinforcing stimuli. One explanation for the intake of high amounts of food in binge eating disorder (BED) is a deviant valuation process. Valuation of food stimuli is supposed to influence approach or avoidance behaviour towards food. Focusing on self-reported and indirect (facial electromyography) valuation process, motivational aspects in the processing of food stimuli were investigated.

**Methods:**

We compared an overweight sample with BED (BED+) with an overweight sample without BED (BED-) and with normal weight controls (NWC) regarding their self-reported and indirect (via facial electromyography) valuation of food versus non-food stimuli.

**Results:**

Regarding the self-reported valuation, the BED+ sample showed a significantly stronger food-bias compared to the BED- sample, as food stimuli were rated as significantly more positive than the non-food stimuli in the BED+ sample. This self-reported valuation pattern could not be displayed in the indirect valuation. Food stimuli evoked negative indirect valuation in all groups. The BED+ sample showed the plainest approach-avoidance conflict marked by a diverging self-reported (positive) and indirect (negative) valuation of food stimuli.

**Conclusions:**

BED+ showed a deviant self-reported valuation of food as compared to BED-. The valuation process of the BED+ sample seems to be characterized by a motivational ambivalence. This ambivalence should be subject of further studies and may be of potential use for therapeutic interventions.

## Introduction

Food stimuli are omnipresent in the western world. On the way to work, one smells freshly baked bread passing a bakery, train stations provide a plethora of food advertisements, in public transport people eat breakfast, and at work one is offered delicious birthday cake. Food is essential for survival and represents a naturally primary reinforcing stimulus which triggers eating behaviour. However, in the absence of physiological need, this highly adaptive mechanism is undesirable. Individuals with binge eating episodes often alternate restrained eating behaviour and binge eating episodes. The complex interplay of valuation, decision-making, and cognitive control processes may be compromised (see [1,2] for a comprehensive review), which again affects eating behaviour. The present study focuses on the valuation process, which is considered an important and cue-elicited process, influencing subsequent decisions and approach or avoidance behaviour [3]. Regarding eating behaviour, valuation can be aversive or appetitive, reflecting a motivational or emotional state. A disturbance of the valuation process can be operationalized through a hypo- or hypersensitivity towards rewarding (food) stimuli [1,4]. The most common dimensions in stimuli evaluation are valence and arousal, whereas especially for food stimuli several further dimensions, such as palatability, wanting and liking, have been established.

The present study seeks to elicit the valuation process regarding food stimuli in individuals with binge eating disorder (BED). This disorder is characterized by regular binge eating episodes, i.e. eating a large amount of food in a certain amount of time accompanied with the feeling of loss of control. The occurrence of regular binge eating episodes suggests that the valuation process of food stimuli in BED might be different from individuals without BED. Studies analysing the processing of food stimuli in individuals with eating disorders (for comprehensive reviews see: [5–7]) have predominantly investigated anorexia and bulimia nervosa, while studies on BED are scarce. Initial brain imaging and eye tracking data indicate that in response to food stimuli BED patients show a higher arousal rate, a concurrent motor planning to start eating, a higher sensitivity to reinforcement, and a higher reward sensitivity than samples without BED [8–12].

The few studies focusing more in depth on the valuation process of individuals with BED can be differentiated in (1) studies using self-reported data, conscious valuation of food stimuli by applying visual analogue scales or rating categories and (2) studies using more indirect psychophysiological data to assess the automatic contribution of this process [13–15]. The available studies regarding the self-reported valuation of food stimuli found either no difference, compared to controls [8,11,12], or inconclusive results. For example, Drobes et al. [16] described that food-deprived individuals and individuals scoring high on binge eating scales rated food stimuli as significantly more pleasant than a non-food-deprived and restrained group. Nevertheless, these samples showed a significantly higher startle reflex, which indicates an aversive response. The authors [16] emphasized this ambivalence, assuming the concurrent activation of opposing motivational circuits. Svaldi et al. [17] examined BED patients and overweight controls, who were shown high-caloric and low-caloric food stimuli while electroencephalographic (EEG) and other psychophysiological data (facial electromyography (EMG), electrodermal activity, electrocardiogram and pulse) were recorded. All participants rated high-caloric food stimuli more palatable than low-caloric food stimuli. Compared to the controls, the EEG data of the BED group showed an increased late positive potential and slow positive wave for high-caloric stimuli, which was interpreted as an indicator of craving. Regarding the psychophysiological data, the only group difference in the high-caloric condition was a lower skin conductance in the BED group compared to the control group. The authors [17] interpreted this as the soothing effect of food. Activation of the corrugator supercilii muscle, which indicates negative valuation, was higher for high-caloric versus low-caloric food across both groups. Finally, Schag et al. [9], investigating an overweight sample with and without BED as well as normal weight controls, found the BED sample to rate the food stimuli as more pleasant than the other groups. In summary, all mentioned results display a highly heterogenic picture and diverging results looking at self-reported or indirect psychophysiological measures.

To our knowledge, except for the study of Svaldi et al. [17], no study thoroughly investigated food valuation in overweight individuals with and without BED using facial EMG. Facial EMG offers a good measure to investigate the indirect and less subjectively driven processes of valuation [15,18–20]. Furthermore, the activation of the corrugator supercilii (CS) denotes aversive valuation, whereas the zygomaticus major (ZM) denotes appetitive valuation [15,21,22]. These muscle activations are elicited rapidly (300–400 ms after stimulus presentation), involuntarily and hard to suppress [15] and highly selective [21].

In the present study—to separate self-reported and indirect valuation processes—rating scales (self-reported) and facial EMG (indirect) were used to measure motivational aspects of food stimulus processing. Whereas Svaldi et al. [17] only compared overweight individuals with and without BED, we additionally added a normal weight control group and also used non-food control stimuli. So far, no study has compared the self-reported versus the indirect valuation of food and non-food stimuli in BED. The investigation of food stimuli valuation in overweight individuals with and without BED will provide valuable information regarding food stimuli as disorder-relevant stimuli and can thus contribute to find further processing differences between individuals with BED and individuals without BED. Finally, all of these results will contribute to a better understanding of motivational aspects regarding the valuation of food in BED. This can be crucial for the progression of therapeutic interventions regarding the reduction of binge eating episodes.

Therefore, we conducted a cross-sectional rating scale and facial EMG study with three groups: (1) overweight participants with BED, (2) age- and weight-matched overweight participants without BED, and (3) age-matched normal-weight controls. To get an insight into the self-reported (rating scales) and indirect (facial EMG) valuation process of food stimuli in individuals with BED we tried to answer the following questions: 1. Are there significant correlations between self-reported and indirect valuation? 2. Do participants with BED evaluate food stimuli differently in valence, palatability, wanting and liking than the two control groups? 3. Is there a difference between the self-reported and the indirect valuation measures in the different samples?

## Materials and Method

### Participants

Seventy-six participants were recruited via email and flyers. To eliminate the influence of gender differences and taking into account the differences in prevalence, only women were included. Because of rather noisy EMG data, 15 out of 76 participants had to be excluded (for details see *EMG recordings and processing*). Participants were assigned to three different groups: (1) overweight participants (body mass index, BMI≥ 26 kg/ m^2^) with BED according to DSM-IV (BED+), (2): overweight controls without BED, matched with the BED+ sample according to body mass and age (BED-), and (3) normal-weight healthy controls (18.5 kg/ m^2^ < BMI < 25 kg/ m^2^), age-matched to the overweight samples (NWC). The cut-off at a BMI of 26 kg/ m^2^ was chosen to get a clear-cut group separation between the samples. The inclusion of one participant in the NWC sample with a BMI of 25.5 kg/m^2^ did not influence any of the results. Exclusion criteria for all samples comprised insufficient knowledge of the German language, incorrectable vision, pregnancy or lactation, any somatic disorder influencing weight or eating behaviour, intake of any psychotropic medication except antidepressants, and the following mental disorders according to DSM-IV: psychosis, bipolar I disorder or substance dependence, and any eating disorder in both control groups.

### Diagnostic instruments

All participants were examined by trained clinicians regarding the diagnosis of BED, ascertained by the German version of the structured clinical interview Eating Disorder Examination (EDE, [23]) and other diagnoses, assessed via the application of the structured clinical interview for DSM-IV (SCID-I, [24]). Depressive symptoms were assessed with the German version of the Beck Depression Inventory (BDI II, [25]). Higher values point to depression with a cut-off value of 13 for a mild and 20 for a moderate depression [26]. State mood was assessed using the 19 items of the Actual Mood Scale with higher values indicating negative mood (ASTS, [27]). Trait and state food craving was assessed using the German Version of the Food Cravings Questionnaires (FCQ, [28]) with higher values representing higher craving. The slightly modified Hunger Scales [29] with higher values indicating more hunger, were used as a control for hunger. The intake of contraceptive pills was ascertained.

### Stimuli

The stimulus array consisted of 40 pictures depicting high-caloric food on a plate (e.g. a pizza, spaghetti bolognese) and 40 non-food pictures (e.g. an empty box, several cords), which were closely matched according to colour, brightness, and visual complexity. The stimulus material has been used in previous experimental studies [30–32]. The caloric content of the foods displayed per picture, was high (*M* = 523.9 kcal, *SD* = 253.4) according to independent ratings by two nutritionists.

### Procedure

Participants were tested individually in two sessions. In the first session, they completed diagnostic questionnaires and were interviewed by trained clinicians. Participants fasted the night before the second session. Adherence to the fasting instruction was prompted and blood sugar level was measured before participants received a standardized breakfast (one bread bun with butter and a slice of cheese, jam and a glass of water). After breakfast, participants filled in a questionnaire about their current feeling of hunger and took part in two additional experimental tasks. In these tasks, food-related impulsivity was assessed via eyetracking. Accordingly (one hour after breakfast), EMG electrodes were placed, participants completed questionnaires concerning their current craving (FCQ-s) and mood (ASTS) and were seated at a distance of about 65 cm to the computer screen (19-inch with a resolution of 1280x1024), where the stimuli were displayed. Participants were instructed to keep their heads still and not to speak or to chew.

**Fig 1:**
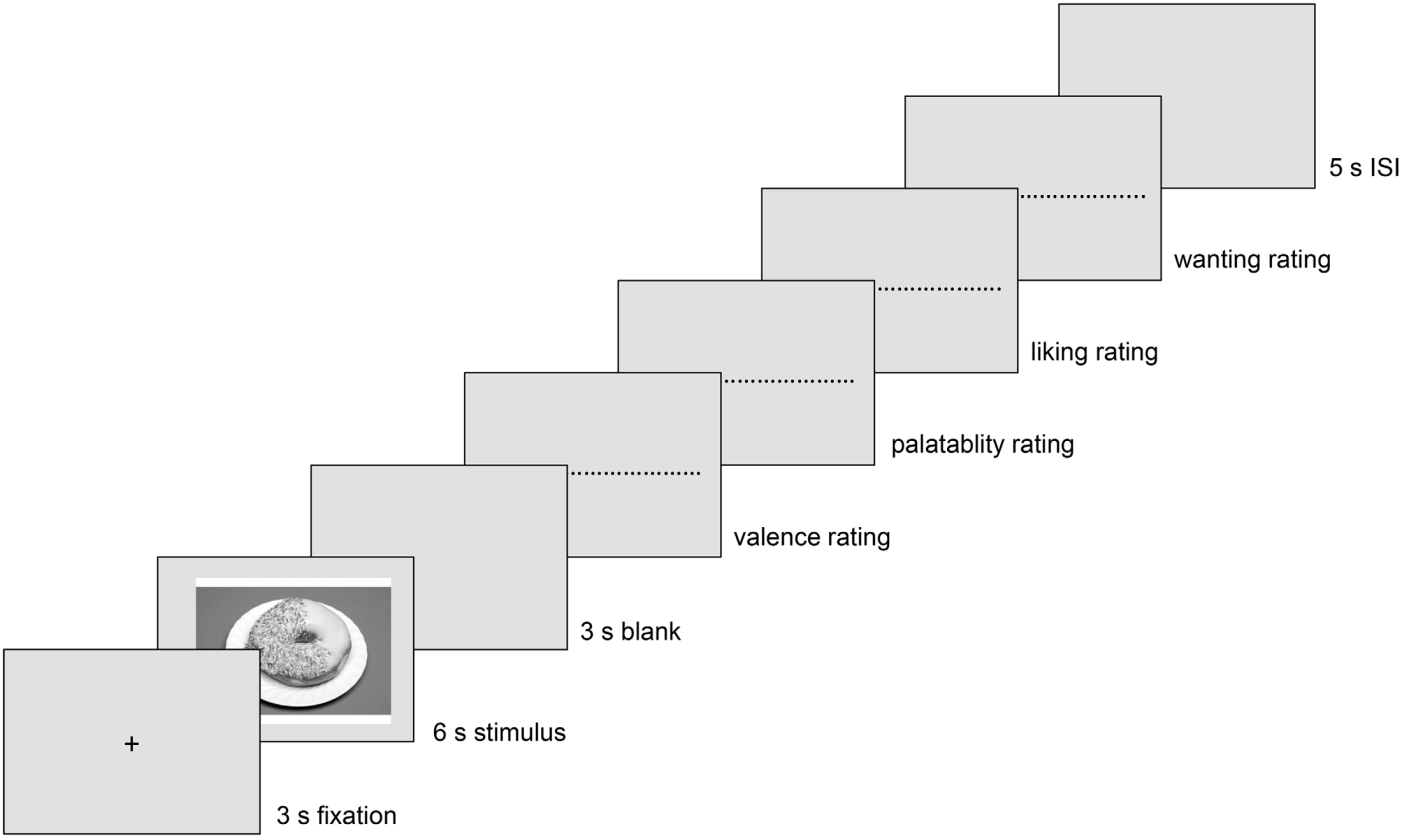
Trial course. ISI = interstimulus-interval.

40 trials with food stimuli and 40 trials with non-food stimuli were presented in a randomized order. The course of each trial can be seen in Fig 1. The valuation of the stimuli was conducted after each stimulus was presented. Participants were to rate them on a Likert-scale ranging from -5 (extremely unpleasant) to +5 (extremely pleasant). For food stimuli they additionally rated on a scale ranging from -5 to +5, how delicious they found the food (“How delicious do you think the presented food is?”), their liking (“How much do you like the presented food in general”) and wanting (“How much would you like to eat the present food right now”). These questions were adopted from Berridge [33].

Participants gave their rating via a standard keyboard. After each trial an inter-trial interval of 5000 ms followed. All participants gave written informed consent to participate in the study and received reimbursement. The ethics committee of the Medical Faculty Tuebingen approved the study (463/2012BO2). The study was conducted according to the Declaration of Helsinki.

### EMG recordings and processing

EMG was recorded using a BrainAmp ExG MR 16 channel system amplifier and analysed using Brain Vision Analyzer Version 2.0 (Brain Products GmbH, Gilching). Four surface electrodes with contact area and housing of 4 mm diameter were attached to the left side of the face, two for the ZM and two for the CS, according to the recommendations by Fridlund and Cacioppo [34]. An additional ground electrode was attached to the forehead. The EMG was continuously recorded at a sampling rate of 5000 Hz and offline down-sampled to 2000 Hz. Raw data were filtered using a 20–500 Hz band-pass filter and a 50 Hz notch filter. After rectifying the data and applying a moving average of 125 ms, the time period 1000 ms before and 6000 ms after stimuli onset was segmented in 10 baseline intervals and 60 stimuli intervals of 100 ms each. Although facial EMG is said to occur after the first second of stimuli onset [14], we decided to analyse the whole 6000 ms of stimulus presentation to get detailed information about valuation processes. Artefact rejection was conducted using the min-max criterion. According to the min-max criterion baseline intervals with changes in amplitude over 8 μV were rejected, whereas for the stimuli intervals amplitude changes over a range of 30 μV were rejected. Participants with a rejection rate of more than 20% of trials in one stimuli category (food or non-food stimuli) were excluded from further data analysis (*n* = 15). They did not differ in sample characteristics from the included participants. Finally, averages of each interval were exported from Brain Vision Analyzer.

### Statistical analyses

All statistical analyses were performed using SPSS Version 21.

*Sociodemographic data* were analysed using univariate analysis of variance (ANOVA) or Kruskal-Wallis test. Reliability of the self-reported valuation was computed using Cronbach’s alpha and intercorrelations were computed using Pearson’s product-moment correlation. Correlations between the *self-reported* and *indirect valuation* were analysed with Pearson’s product-moment correlation across the overall sample.

Data of the *self-reported rating* scales were analysed using ANOVA with the group as the independent and the rating as the dependent variable. For the valence dimension, we used a repeated-measure ANOVA with stimulus (food versus non-food) as within-subject factor and group (BED+ versus BED- versus NWC) as between-factor. For significant effects or interactions Bonferroni-corrected post-hoc tests (α-value = .017) were conducted. For the interpretation of significant interactions, a food bias score (Δ valence food—valence non-food stimuli) was used. Pearsons’s product-moment correlations between the self-reported valuation and craving were reported for each group, including bootstrapped relationships estimates taking into account small sample size.

We also conducted a *t*-test comparing BED+ participants (eating disorder sample) with the BED- and the NWC group as one sample without eating disorder regarding the self-reported valuation dimensions valence, palatability, liking and wanting.

*Facial EMG data* were examined regarding group differences in the mean baseline activation, but no differences were found. For further data analysis we computed values of relative change to the baseline (Δ_i_ = mean activation in each 100 ms interval—mean baseline activation). To facilitate the interpretation of the results, EMG data during the stimuli presentation were reduced by subsuming the data into twelve time intervals of 500 ms each. As distinct facial reactions take about 300–400 ms to develop [19,35] we decided to use 500 ms intervals. EMG data were analysed using 2x12x3 design with stimulus (food versus non-food) and time interval (one to twelve) as within-subject factors and group (BED+ versus BED- versus NWC) as between-subject factor. In case of sphericity violation, Greenhouse–Geisser corrected values are reported. Bonferroni-corrected post-hoc tests were used to identify the source of interactions between the within factors.

## Results

### Sociodemographic data

Sample characteristics are displayed in Table 1. No differences between the groups were found regarding the intake of oral contraception, *X*^*2*^(2) = .3, *p* = .84, Cramer’s *V* = .076. There were no group differences regarding the blood sugar level before breakfast, *F*(2, 58) = 1.7, *p* = .197, *η*_*p*_^*2*^ = .055. We also checked for any group differences in reported hunger after the standardized breakfast, which was not the case, *H*(2) = .5, *p* = .761, *r* = .077.

**Table 1:** Means (SD) [range] of sociodemographic data, psychopathology and food craving of all participants.

	BED+ (n = 16)	BED- (n = 23)	NWC (n = 22)	Test statistics (*H* or *F*)	*p*
**Age (years)**	32.1 (11.6) [18.0–53.00]	33.3 (13.2) [20.0–64.0]	35.2 (12.3) [21.0–60.0]	1.3	.534
**BMI (kg/m**^**2**^**)**	34.7 (5.1)^b^ [26.4–45.9]	33.4 (4.5)^b^ [26.7–45.8]	22.1 (1.6)^a^ [19.1–25.5]	62.5	< .001
**Depression**^1^	15.7 (8.5)^c^ [3.0–33.0]	6.6 (5.6)^b^ [0–21.0]	2.3 (1.9)^a^ [0–6.0]	27.6	< .001
**Negative mood**^2^	65.8 (12.7)^b^ [38.0–85.0]	50.6 (12.9)^a^ [28.0–75.0]	58.7 (15.6) [34.0–101.0]	5.6	.006
**Craving (trait)**^3^	145.1 (23.2)^b^ [108.0–201.0]	85.2 (30.6)^a^ [45.0–161.0]	69.6 (16.5)^a^ [44.0–95.0]	47.5	< .001
**Craving (state)**^3^	34.3 (18.6)^b^ [15.0–70.0]	20.2 (7.0)^a^ [8.0–37.0]	20.5 (9.2)^a^ [15.0–47.0]	6.7	.035

*Note*: BED+ = overweight participants, BED- = overweight controls without BED, NWC = normal weight healthy controls, BMI = Body Mass Index,

^1^: measured with the Beck Depression Inventory,

^2^: measured with the Actual Mood Scale,

^3^: measured with Food Cravings Questionnaire,

^a, b, c^ = group means with different subscripts differ from each other.

### Correlation of self-reported and indirect valuation

Cronbach’s alpha values and intercorrelation between the different valuation scales for food stimuli are reported in Table 2. For the non-food stimuli the valence scale reached a Cronbach’s alpha of α = .928.

**Table 2:** Reliabilities and Pearson’s product-moment correlations between the self-reported valuation dimensions.

	Valence	Palatability	Liking	Wanting
**Valence**	*(*.*953)*	.957**	.716**	.806**
**Palatability**		*(*.*948)*	.726**	.817**
**Liking**			*(*.*976)*	.555**
**Wanting**				*(*.*930)*

*Note*: Cronbach’s alpha is presented in the diagonal.

** signifies *p*-values< .01.

Each of the 12 *EMG* data intervals was correlated with the rating of valence for food and non-food stimuli separately for each muscle. The aim was to test the relation between the self-reported and the indirect measures. All correlations are presented in Table 3.

**Table 3:** Pearson’s product-moment correlations between the intervals of the indirect valuation and the self-reported valence rating.

**Corrugator supercilii**												
	*500*	*1000*	*1500*	*2000*	*2500*	*3000*	*3500*	*4000*	*4500*	*5000*	*5500*	*6000*
**Food stimuli**												
Valence	*-*.*00*	*-*.28*	*-*.24*	*-*.*20*	*-*.*19*	*-*.*19*	*-*.*20*	*-*.26*	*-*.24*	*-*.24*	*-*.23*	*-*.26*
Palatability	.*04*	*-*.25*	*-*.23*	*-*.*20*	*-*.*18*	*-*.*20*	*-*.22*	*-*.28*	*-*.25*	*-*.24*	*-*.24*	*-*.28*
Liking	.*14*	*-*.*21*	*-*.*19*	*-*.*16*	*-*.*17*	*-*.*15*	*-*.*17*	*-*.22*	*-*.28*	*-*.26*	*-*.23*	*-*.26*
Wanting	.*07*	*-*.*20*	*-*.*18*	*-*.*16*	*-*.*16*	*-*.*19*	*-*.*19*	*-*.*16*	*-*.*13*	*-*.*12*	*-*.*13*	*-*.*17*
**Non-food stimuli**												
Valence	*-*.*01*	*-*.*01*	*-*.*04*	*-*.*04*	.*03*	*-*.*05*	*-*.*02*	*-*.*06*	*-*.*12*	*-*.*16*	*-*.*20*	*-*.*11*
**Zygomaticus major**												
	*500*	*1000*	*1500*	*2000*	*2500*	*3000*	*3500*	*4000*	*4500*	*5000*	*5500*	*6000*
**Food stimuli**												
Valence	*-*.*02*	*-*.*09*	*-*.*11*	*-*.*03*	.*04*	*-*.*01*	*-*.*02*	*-*.*12*	*-*.*04*	*-*.*06*	*-*.*09*	*-*.*12*
Palatability	.*03*	*-*.*03*	*-*.*09*	*-*.*05*	*-*.*00*	*-*.*02*	*-*.*01*	*-*.*10*	*-*.*05*	*-*.*05*	*-*.*09*	*-*.*14*
Liking	.*20*	.*10*	.*01*	.*01*	.*-06*	.*-11*	*-*.*06*	.*-05*	.*-02*	*-*.*01*	*-*.*05*	*-*.*11*
Wanting	*-*.*01*	*-*.*05*	*-*.*12*	*-*.*03*	.*03*	.*02*	*-*.*01*	*-*.*06*	*-*.*02*	*-*.*04*	*-*.*06*	*-*.*07*
**Non-food stimuli**												
Valence	*-*.28*	*-*.*16*	*-*.*12*	.*08*	.22*	.39**	.35**	.*19*	.23*	.23*	.*20*	.*13*

*Note*:

* signifies *p*-values< .05;

** signifies *p*-values< .01.

### Self-reported valuation

Univariate ANOVAs regarding group differences in the valuation of food stimuli revealed no main effect regarding the factor group concerning the valuation, except for the wanting scale (see Table 4).

**Table 4:** Means (SD) [range] of self-reported valuation regarding food stimuli.

Food stimuli	BED+ (n = 16)	BED- (n = 23)	NWC (n = 22)	*F*	*p*	*η*_*p*_^*2*^
Palatability	1.7 (1.8) [-2.7–4.7]	1.0 (1.6) [-1.6–3.4]	1.3 (1.3) [-2.1–3.7]	1.0	.382	.033
Liking	0.3 (2.7) [-4.7–4.8]	-.8 (2.3) [-5.0–3.1]	-1.2 (2.2) [-4.7–2.7]	2.2	.123	.070
Wanting	2.4 (1.3)^b^,* [-0.9–3.8]	1.2 (1.3)^a^ [-1.7–3.1]	1.4 (1.6)^+^ [-2.9–3.3]	3.3	.045	.101

*Note*: BED+ = overweight participants with BED according to DMS-IV, BED- = overweight controls without BED, NWC = age-matched, normal weight healthy controls. Rating ranges between -5 and +5.

^a, b^ = group means with different subscripts differ from each other.

^+^, * = trend to significant differences between data with superscript + and superscript *.

**Fig 2:**
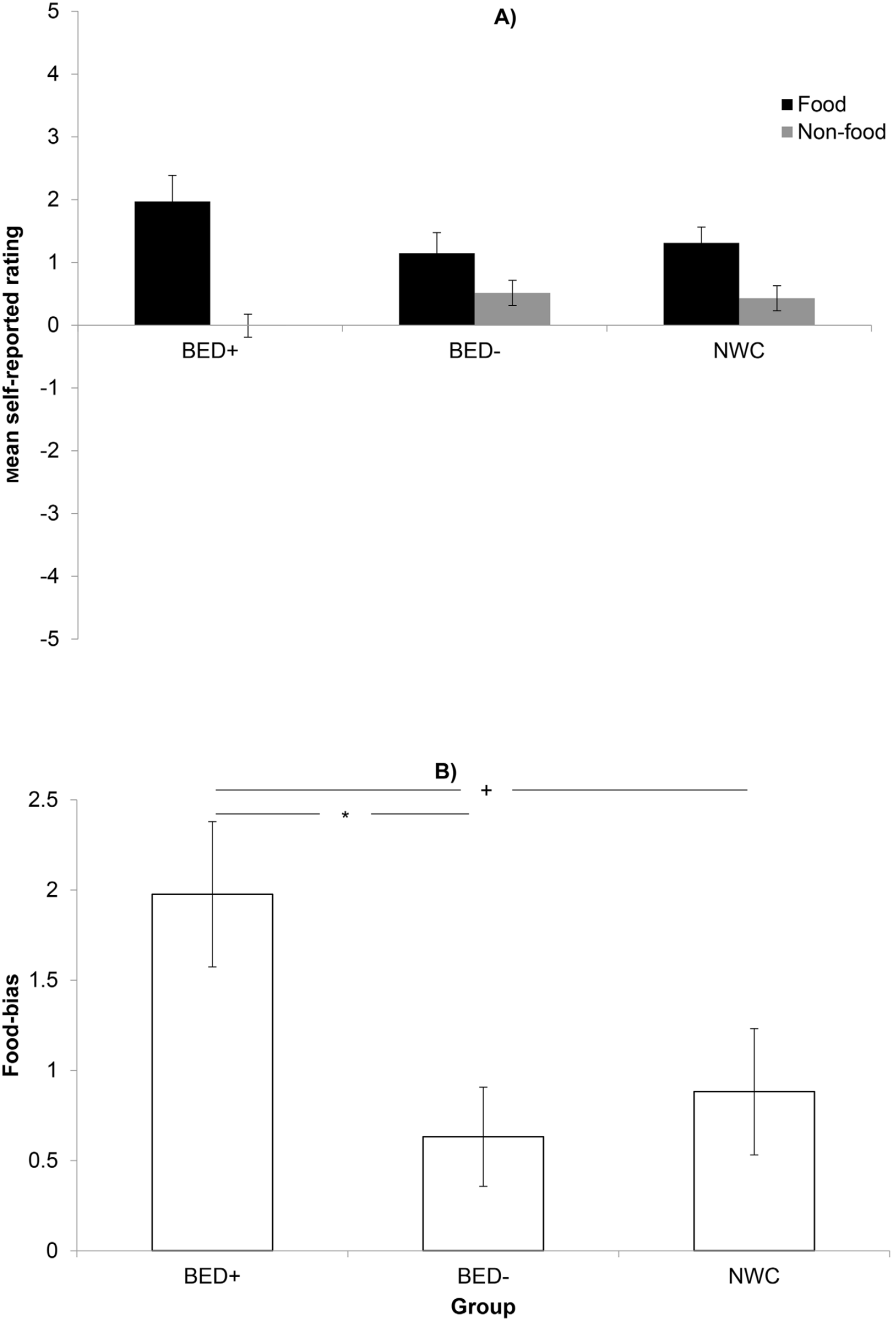
Mean self-reported valence of food versus non-food stimuli in all three groups. BED+ = overweight participants with BED according to DMS-IV, BED- = overweight controls without BED, NWC = age-matched, normal weight healthy controls. * indicates significant differences after Bonferroni correction (*p*< .017), + indicates significant differences with p-values< .05.

Testing for motivational aspects regarding the valence of the food stimuli compared to the valence of the non-food stimuli, the repeated measures ANOVA revealed a main effect for stimulus, *F*(1,58) = 34.9, *p*< .001, *η*_*p*_^*2*^ = .376. A significant interaction between group and stimulus was found, *F*(2,58) = 4.0, *p* = .024; *η*_*p*_^*2*^ = .121, which was due to a stronger food bias effect in the BED+ group as compared to the BED- group (Fig 2). There was no main effect regarding the factor group, *F*(2,58) = .1. *p* = .885, *η*_*p*_^*2*^ = .004.

When comparing the group with eating disorder (BED+) to the groups without eating disorders (BED- and NWC), there was a significant group effect regarding the liking, *t*(59) = 2.0, *p* = .049 *r* = .253, and the wanting rating, *t*(59) = 2.5, *p* = .014 *r* = .314,. The BED+ group rated food stimuli higher on the liking (*M*_*liking*_ = 1.3, *SD*_*liking*_ = 2.7) and wanting (*M*_*wanting*_ = 2.4, *SD*_*wanting*_ = 1.3) scale than the non-ED group (*M*_*liking*_ = -1.0, *SD*_*liking*_ = 2.2; *M*_*wanting*_ = 1.3, *SD*_*wanting*_ = 1.4).

Correlation between self-reported valuation and the FCQ-s were analysed for each group (BED+, BED- and NWC). The BED+ sample showed a significant correlation between FCQ-s and the liking scale, *r* = .586, *p* = .022, bootstrap bias = -.005, *SE* = .14, 95%-confidence interval = .250- .820. For the BED- sample there were no significant correlations found. In the NWC sample the liking scale was significantly correlated with the FCQ-s score, *r* = .534, *p* = .011, bootstrap bias = -.019, *SE* = .162, 95%-confidence interval = .131- .771.

### Indirect valuation

**Fig 3:**
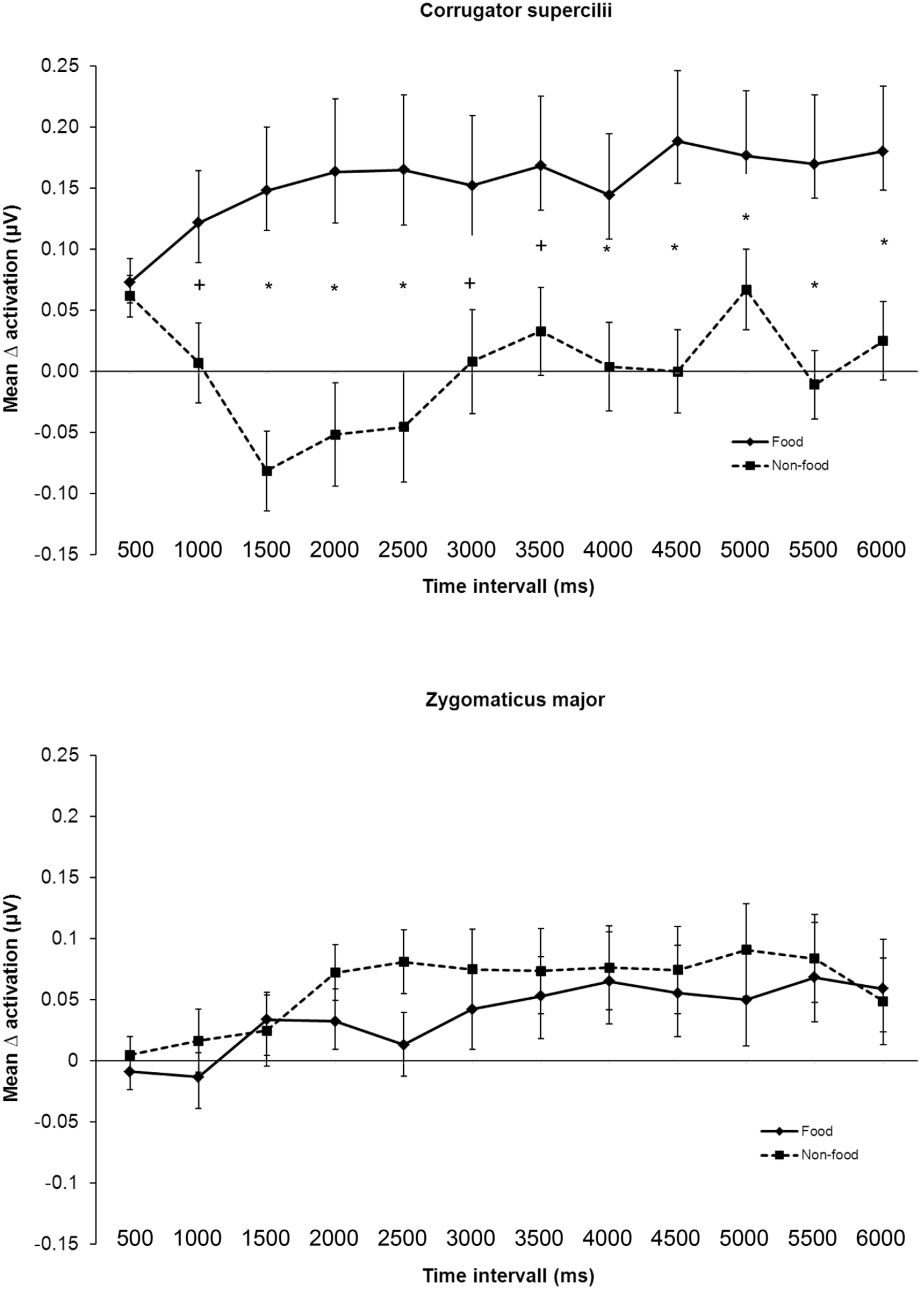
Time course of the mean Δ activation and standard errors for the corrugator supercilii and the zygomaticus major activation over all groups. * indicates significant differences after Bonferroni correction (*p*< .004), + indicates significant differences with *p*-values< .05.

The overall course of muscular response across all three groups can be seen in Fig 3.

Regarding the CS activation the repeated measures ANOVA revealed a significant main effect for stimulus, *F*(1, 58) = 14.9, *p*< .001, *η*_*p*_^*2*^ = .204, but not for group, *F*(2, 58) = 2.7, *p* = .08, *η*_*p*_^*2*^ = .084. Furthermore, there was a significant interaction between time interval and stimulus, *F*(4.9, 285.7) = 3.7, *p* = .003, *η*_*p*_^*2*^ = .06. Bonferroni-corrected post-hoc tests (α = 0.004) revealed significant differences between the CS activation for food versus non-food stimuli over time. In contrast, for ZM no significant main or interacting effects could be found. Fig 3 displays the time course of the CS and the ZM activation.

## Discussion

The present study is one of the first to investigate the valuation of food versus non-food stimuli in individuals with BED and to integrate both self-reported and indirect valuation data. The aims of the study were: 1. to obtain initial insights into the self-reported and indirect valuation, to identify group differences in motivational aspects by comparing the valuation of an overweight BED group (BED+) with a BMI-matched control group without BED (BED-) and a normal-weight control group (NWC) 2. to explore possible relations between the self-reported and the facial EMG measurement. The self-reported valuation was measured via rating scales, whereas the indirect valuation was measured via facial EMG.

A small significant negative correlation between the self-reported valuation dimensions valence, palatability and liking and the CS activation was found for food stimuli. This indicates that individuals, who rate the food stimuli more positively, palatable or likable, display a lower CS activation and vice versa. In contrast, ZM activation correlated significantly positively with the self-reported valence rating in late time intervals for non-food stimuli. First, these results suggest that self-reported and indirect measurements of valuation processes are not interchangeable. This strengthens the need of method combination when investigating valuation processes. Second, it might also strengthen the assumption that valuation processes are highly complex and influenced by several factors (e.g. social desirability, craving). The fact of non-interchangeability of self-reported valuation and all kind of indirect physiological data was also stated by Drobes et al. [16]. Testing the relationship between facial reactivity and self-reported reward judgments of food pictures, Soussignan et al. [36] did not find predictive power between these two measures. Third, negative correlations for food stimuli also reflect the later discussed divergence between self-reported and indirect valuation.

Self-reported valuation results revealed a significant higher food bias in the BED+ compared to the BED-sample, whereas the NWC sample lays in-between. This effect can be interpreted as evidence that individuals with BED perceive food stimuli as exceptional stimuli with a motivational significance as well as NWC, but may have less ability to control motivational approach. This assumption is supported by a larger food bias for individuals with BED compared to NWC, which reaches significance when applying a p-value of < .05. This food bias can be one factor, contributing to the binge eating pathology. Further, individuals with BED rated food stimuli higher on the wanting scale than the BED- group. This result matches the significantly higher score on state food craving in the BED+ group. There are also several studies showing behavioral inhibition deficits in individuals with BED [9,37,38].

Analysis of facial EMG data showed that food stimuli evoked significantly higher aversion than non-food stimuli in all groups. The BED+, the BED- and the NWC samples reported food pictures as significantly more positive than non-food stimuli, whereas all groups displayed a higher (indirect) activation of the CS in response to food stimuli than to non-food stimuli. Hence, there seems to be ambivalence between the self-reported and indirect valuation process: although food stimuli are rated as positive regarding the self-reported data, the indirect valuation via facial EMG is negative. One possible reason for the conflicting results between self-reported and indirect valuation could be that subjects were sated, which explains an automatic avoidance response regarding the facial EMG. Nevertheless, whereas facial EMG in early time intervals represent mainly automatic processes, later time intervals might reflect automatic as well as controlled valuation processes. Especially in the BED+ group with higher trait and state craving pure automatic avoidance could be doubted and the influence of partly controlled avoidance processes (e.g. negative consequences of binge-eating) seems to be likely. A second possible explanation could be that the stimulus material was not perceived as very attractive, which was also indicated by the only slightly positive self-reported valence rating. A third explanation, referring to the similar results of Svaldi et al. [17], would be an aversion towards specifically high-caloric food stimuli. Whereas for the BED+ sample the aversion could be easily explained—as binge eating is often connected with negative feelings like shame or guilt—the question remains why individuals in the BED- and NWC group should feel aversion towards high-caloric food stimuli. Hawk et al. [39] found a higher startle reflex in subjects without eating disorder, strongly craving food. These results support the hypothesis that beside the eating disorder diagnosis other characteristics such as craving seemed to be of additional importance, when investigating the valuation of food stimuli. Nevertheless, our data revealed a higher craving only for the BED+ and not for the BED- and NWC group. Drobes et al. [16] found as well a divergence between self-reported and indirect valuation: food-deprived participants and participants showing binge-like eating behaviour rated food stimuli more positive as well as showing aversive valuation patterns, operationalized through a higher startle response.

The dissociation of self-reported and facial EMG data might be driven by a motivational conflict between approach and avoidance. The approach-avoidance conflict together with deficient cognitive control and behavioural inhibition deficits could be one influential factor for the deviant eating behaviour in individuals with BED. Strong adverse approach behaviour towards food stimuli (i.e. in the absence of physiological need) require efficient cognitive control and inhibition capacities to result in non-deviant eating behaviour. We found no effects of group, stimulus or time interval examining the ZM activation.

When interpreting the results of this study, it is necessary to bear in mind some of the limitations: after the first data inspection, the sample was reduced (*n* = 61). Further, only high-caloric food pictures and non-food stimuli were used. On the one hand, the comparison of food versus non-food stimuli allows the investigation of food-specific differences in the valuation process. On the other hand, effects within the processing of different food stimuli (e.g. high- versus low-caloric food) or other rewarding non-food stimuli (e.g. money) cannot be revealed. The fact, that stimulus material was already known to all participants, although in a different experimental context, might have influenced the valuation process in all groups. Habituation might have reduced the effects to the here presented ones. The inclusion of other characteristics regarding the stimulus material like energy density, individual preference or eating habits could give further interesting insights. Additionally, comparability of studies investigating the processing of food stimuli would be improved by using of a common picture sample. A first attempt to standardise the stimulus material, as it is already the case with emotional pictures, was started recently [40]. Moreover, only EMG was applied to measure the indirect evaluation and it would have been interesting to ascertain data from different, simultaneously applied methods such as EEG, startle reflex, or skin conductance. Nevertheless, the inclusion of further indirect measures could result in further inconsistencies between different measurements. An important supplement would be the integration of process-related behavioural data. In regular approach-avoidance paradigms this has been already done by observing respective behaviour, such as movements towards or away from a stimulus (e.g. [41]). Another aspect is the homeostatic state of subjects. In our study—as well as in the study of Svaldi et al. [17]–subjects were sated, which might be responsible for the rather small effects. There is evidence that the comparison of fasted and satiated individuals can give further insights in phenotypic differences [4]. Then, the argument always remains that, while pictures of food were presented, the evaluation of food has many more levels of perception than just sight, for example smell and taste. Lastly, it would have been interesting to assess other addictive behaviours beside substance addiction, like compulsive buying or internet addiction.

To conclude, this study is the first to compare a BED+ sample with a BED- and a NWC sample looking at their self-reported and indirect valuation of food versus non-food stimuli. It could be shown that there is a deviant self-reported valuation of food stimuli in overweight participants diagnosed with BED compared to non-eating disordered overweight individuals. We revealed motivational ambivalence in the BED+ sample, marked by valence differences in the self-reported and the facial EMG data. These results confirm the assumption that individuals with BED+ represent a distinct phenotype in the obesity spectrum [4,42,43]. Regarding the treatment of individuals with BED, the putative ambivalence should be considered in therapy. Assuming that automatic as well as controlled valuation processes are relevant for the eating behaviour, one could suppose that the reduction of approach tendencies might contribute positively to the binge eating disorder pathology. Cognitive interventions (e.g. reappraisal taking into account short- and long-term aspects of excessive food ingestion, situation analysis to find triggers for craving) as well as behavioural interventions (e.g. exposition exercises with food stimuli, training of anti-craving strategies, stimulus control and situation control) could influence approach behaviour as they promote controlled processing of food stimuli and reduce automatic approach behaviour. In a recently published study a cognitive bias modification programme for participants with high-level craving reduced self-reported food-craving tendencies and approach bias turned into an avoidance bias towards food stimuli regarding data of a four week follow-up [44]. Another study with normal-weight females resulted in inconclusive results regarding the effectiveness of an approach-avoidance training [45]. Directions for research should be the presentation of additional low-caloric food stimuli and rewarding non-food stimuli. Further, assuming that self-reported valuation represents a long-term representation of food stimuli, which might be also one explanation for the divergence between self-reported and indirect valuation, development of methods to change this long-term representation might be promising. Another important step would be to investigate the behavioural consequences of these valuation processes through the inclusion of evaluation data in more naturalistic designs, including virtual reality or ecological momentary assessment. In addition, it would be interesting to compare fasted to satiated groups. Lastly, the motivational ambivalence in the BED+ sample should be subject to further studies. Further results confirming this motivational ambivalence could enable the development of therapeutic interventions.

## Abbreviations

ANOVA: analysis of variance
ASTS: Actual Mood Scale
BDI-II: Beck Depression Inventory
BED: binge eating disorder
CS: corrugator supercilii
DSM: Diagnostic and Statistical Manual of Mental Disorders
EEG: electroencephalography
EDE: Eating Disorder Examination
EMG: electromyography
FCQ: Food Craving Questionnaire
NWC: normal weight healthy controls
SCID: Structured Clinical Interview for DSM-IV
SD: standard deviation
SE: standard error
ZM: zygomaticus major
